# A Prospective Study of Early Radiation Associated Cardiac Toxicity Following Neoadjuvant Chemoradiation for Distal Esophageal Cancer

**DOI:** 10.3389/fonc.2020.01169

**Published:** 2020-08-06

**Authors:** Aidan M. Burke, Celine Yeh, Sunnie Kim, Peter Bergquist, Pranay Krishnan, Ana Barac, Monvadi B. Srichai, Keith Unger

**Affiliations:** ^1^Department of Radiation Medicine, MedStar Georgetown University Hospital, Washington, DC, United States; ^2^Lombardi Comprehensive Cancer Center, MedStar Georgetown University Hospital, Washington, DC, United States; ^3^Department of Radiology, MedStar Georgetown University Hospital, Washington, DC, United States; ^4^Department of Cardiology, MedStar Georgetown University Hospital, Washington, DC, United States

**Keywords:** radiation, cardiac damage, early detection, cardiac imaging, esophageal cancer

## Abstract

**Purpose:** This study aimed to prospectively evaluate the early effects of radiation on cardiac structure and function following neoadjuvant chemoradiation for distal esophageal cancer.

**Methods and Materials:** Patients with non-metastatic esophageal cancer who were suitable for tri-modality therapy with concurrent chemoradiotherapy followed by esophagectomy were enrolled. Cardiac magnetic resonance imaging (CMR) was obtained at baseline and 3–5 months following completion of chemoradiation. Standardized myocardial segmentation was used to identify regions on post-treatment CMR with new T2 signal or late gadolinium enhancement (LGE). Pre and post-treatment cardiac function was assessed with quantitative end points including left ventricle end-systolic volume (LSESV). Serum biomarkers of cardiac damage including troponin I, CRP, and BNP were collected at baseline and time of follow-up CMR.

**Results:** A total of 11 patients were enrolled from 2016 to 2018. Patients had clinical stage T2 (18%) and T3 (82%) disease with clinical N0 (27%) and N1 (73%) nodal stage. All patients completed baseline CMR and completed chemoradiotherapy. One patient did not complete follow-up CMR or serum biomarkers and was excluded from the analysis. The median time from completion of chemoradiation to follow-up CMR was 3.9 months. Three out of 10 patients (30%) developed new structural findings of myocardial fibrosis and/or reversible ischemia involving the basal and mid-inferior and inferoseptal walls. In these three patients, the LVESV was significantly increased from baseline following radiation. There were no differences in other quantitative end points or serum biomarkers between the patients with myocardial damage and those without.

**Conclusions:** Our study is the first to our knowledge to prospectively demonstrate radiation associated structural and functional heart damage as early as 3 months following neoadjuvant chemoradiation for distal esophageal cancer. Given the early onset of this subclinical heart damage, strategies should be developed to identify patients at risk for future clinically significant heart toxicity.

## Introduction

Increasing numbers of patients with esophageal cancer are being treated with combined modality therapy including chemotherapy and radiation. Due to improvements in outcomes, cancer survivorship has increased and there is a growing focus on long-term treatment-related toxicity. The association of increasing exposure to ionizing radiation with long-term cardiac toxicity is well-established in breast cancer (1, 2), lymphoma (3, 4), and lung cancer (5–7). Cardiac toxicities include pericarditis, ischemic cardiovascular disease, cardiomyopathy, valvular dysfunction, clinical heart failure, and arrhythmias. The latency period for radiation-induced heart disease may range from months to many years and is dependent on radiation dose, age, preexisting cardiovascular disease, traditional risk factors, and concurrent chemotherapy. Cardiovascular toxicities from radiation can interfere with optimal cancer management, decrease quality of life, and affect overall survival (5, 6, 8, 9).

Although neoadjuvant chemoradiation offers a significant survival advantage in patients with resectable esophageal cancer, large volumes of normal tissues are frequently irradiated to cover sites of tumor spread, potential lymph node involvement, and target motion (10). Cardiac dose may be alarmingly high in distal esophageal cancer in particular. Given the generally higher cardiac radiation doses that are expected in the treatment of esophageal cancer, one would expect significant cardiac morbidity and mortality following treatment. Indeed, recent analyses of the SEER database have demonstrated that that the use of radiation therapy for esophageal cancer leads to an increased risk of cardiac death (11, 12). Therefore, esophageal cancer survivors who undergo radiation therapy should be carefully monitored for the development of acute and long-term cardiac toxicity.

Cardiac imaging can play an important role in pretreatment risk assessment, early detection of cardiac injury, and identification of cardiac complications in patients receiving potentially cardiotoxic cancer treatment. Numerous previous reports and meta-analyses have concluded that stress cardiac magnetic resonance imaging (CMR) is a sensitive and specific method of detecting coronary arterial disease, myocardial fibrosis, and cardiac function (13–15). Stress CMR consists of bright blood cine imaging to examine regional left ventricular (LV) wall motion, stress, and rest first pass perfusion imaging to assess for ischemia, and late gadolinium enhancement (LGE) imaging to detect myocardial infarction and/or fibrosis.

American Society of Clinical Oncology (ASCO) cancer survivorship guidelines suggest follow-up cardiac imaging 6–12 months after completion of cancer therapy in patients considered to be at elevated risk for heart failure (16). Similarly, guidelines from the National Comprehensive Cancer Network (NCCN), European Society for Medical Oncology (ESMO) and American Society of Echocardiography (ASE) and European Association of Cardiovascular Imaging (EACVI) recommend post-treatment cardiac surveillance for cancer survivors who receive cardiotoxic therapy (17). While the guidelines uniformly recommend cardiac imaging, the timing, modality, and relevant findings are not uniform across these guidelines and reflect the lack of strong data regarding acute and long-term cardiac toxicity from radiation.

In addition to imaging evaluation of left ventricular ejection fraction (LVEF) and myocardial damage, there has also been considerable interest in biomarkers to identify early cardiac injury such as elevated troponins, B-type natriuretic peptides (BNP), N-terminal T-pro-BNP (NT-proBNP) and C-reactive protein (CRP) (18–20). Collection of serum biomarkers is minimally invasive, relatively cheap, easily interpretable and can be readily repeated. ASCO and the ASE/EACVI include only troponin measurements in their follow-up guidelines. The use of other biomarkers continues to be the subject of investigation and is limited by the inconsistent data on the timing of measurements, thresholds to define toxicity and choice of assay.

While the long-term effects of radiation on the heart have been well-characterized, there is a paucity of data on acute cardiac toxicities. Early identification of cardiotoxicity could lead to better preventative strategies and improved radiation therapy delivery techniques to mitigate clinically significant cardiac toxicity following thoracic radiotherapy. We therefore sought to prospectively collect baseline and acute post-treatment cardiac imaging and biomarkers to correlate early structural and functional changes with clinically significant cardiac outcomes, as well as to identify dosimetric predictors of cardiac toxicity.

## Methods

### Patient Eligibility

This prospective, IRB-approved study enrolled patients seen at the MedStar Georgetown University Hospital who had non-metastatic esophageal cancer and were suitable candidates for tri-modality therapy with concurrent chemoradiotherapy followed by esophagectomy from 2016 to 2018. Patients were required to be at least 18 years old and have histologically proven diagnosis of adenocarcinoma or squamous cell carcinoma of the esophagus or gastroesophogeal (GE) junction. Patients could not have any evidence of metastatic disease, have received prior thoracic radiotherapy or underwent esophagectomy. Eastern Cooperative Group (ECOG) performance status had to be 0–1 with a predicted life expectancy >3 months. Patients could not have a prior history of unstable angina, uncompensated congestive heart failure (CHF), uncontrolled hypertension (systolic > 220 mmHg, diastolic > 120 mmHg), severe mitral or aortic stenosis, advanced atrioventricular (AV) node block without pacemaker, electronically active implants, other non-MR compatible implants, contraindications to gadolinium contrast or acute systemic illness.

### Cardiac Magnetic Resonance Imaging

Magnetic resonance imaging was performed with a 1.5 Tesla MR system using a torso and spine coil in conjunction with electrocardiographic gating. Cardiac MRIs were obtained at baseline and 3–5 months following completion of chemoradiation. The imaging protocol consisted of scout images to identify cardiac axes, black blood double inversion-recovery imaging of the thorax in the axial planes, cine 2D steady-state free precession imaging in standard long-axis planes, and stacked short-axis planes to cover the entire left ventricle, T2-weighted turbo spin echo imaging with fat suppression, dynamic first-pass perfusion imaging at rest and ~90–120 s following the administration of 0.4 mg of regadenoson in conjunction with 0.05 mmol/kg gadolinium contrast injection, and inversion-recovery late gadolinium enhancement imaging 10–15 min following intravenous injection of a total of 0.15 mmol/kg gadolinium contrast with inversion time adjusted to null normal myocardium. Left ventricular volumes and ejection fraction were calculated using cine short-axis planes. Presence and extent of increased T2 signal and/or late gadolinium enhancement were graded using standard 17-segment American Heart Association (AHA) model of the heart (21).

### Treatment Details

Patients underwent simulation with 4D CT to account for respiratory motion. A PET/CT in the treatment position was obtained and fused with the treatment-planning CT. Target volumes were delineated as per the Alliance 80803 study (22). Patients were treated with Intensity Modulated Radiation Therapy (IMRT) or Proton Beam Therapy (PBT) to a total dose of 50.4 Gy in 28 daily fractions to all sites of gross disease. Elective nodal areas were treated to 45 Gy. Concurrent chemotherapy consisted of four to six cycles of intravenous carboplatin and paclitaxel. Patients underwent esophagectomy 4–6 weeks following the completion of radiation therapy.

### Cardiac Contouring

Pre and post-treatment CMR sequences were fused to the treatment-planning CT. Relevant cardiac anatomy including total heart and left ventricle were contoured. Standardized myocardial segmentation per the American Heart Association 17 segment model was used (21). Contours were retrospectively generated by a radiation oncologist and thoracic radiologist. The dosimetric data from the treatment plan was then analyzed with respect to the contoured structures and included mean doses, maximum point doses, and volume receiving 30 Gy (V30).

### Serum Biomarkers

Serum biomarkers were collected at baseline and 3–5 months following completion of chemoradiation. Biomarkers included troponin-I, CRP, BNP, NT-pro-BNP, and CRP.

### Statistics

Pre and post-radiation values for serum biomarkers were compared using paired *t*-test. Dose statistics to the heart, left ventricle and grouped heart segments were compared using paired *t*-test between patients with CMR evidence of myocardial injury and those without. Tests of cardiac function such as left ventricular ejection fraction (LVEF), left ventricular end systolic volume (LVESV), left ventricular end diastolic volume (LVEDV), cardiac output (CO), and systolic volume (SV) were compared using paired *t*-test between those with evidence of myocardial injury and those without. All statistics were performed using SPSS Statistical Software (IBM Corporation, Armonk, NY, USA).

## Results

### Patient and Treatment Characteristics

A total of 11 patients were enrolled from February 2016 to February 2018. The median age was 69 (range 37–80 years) and the majority of patients were male (82%). Patients had clinical stage T2 (18%) and T3 (82%) disease with clinical N0 (27%) or N1 (73%) nodal stage. The majority of patients had adenocarcinoma (*n* = 10) and one patient had squamous cell carcinoma. All patients completed baseline CMR and completed chemoradiotherapy. Ten patients underwent IMRT with photons and one patient was treated with proton beam therapy. One patient demonstrated complete response to neoadjuvant chemoradiation and did not have further therapy while the remainder underwent esophagectomy. One patient did not complete follow-up CMR or serum biomarkers and was excluded from the analysis. The median time from completion of chemoradiation to follow-up CMR and serum analysis was 3.9 months (range 3–5 months). Patient characteristics are shown in Table 1.

**Table 1:** Patient characteristics.

**Age (years)**	
Median	69
Range	37–80
**Sex (%)**	
Male	9 (82)
Female	2 (18)
**Tumor type (%)**	
Adenocarcinoma	10 (91)
Squamous cell carcinoma	1 (9)
**Clinical N stage (%)**	
cT2	2 (18)
cT3	9 (82)
**Stage group (%)**	
IIA	3 (27)
III	8 (73)

### Cardiac Magnetic Resonance Imaging

**Figure 1:**
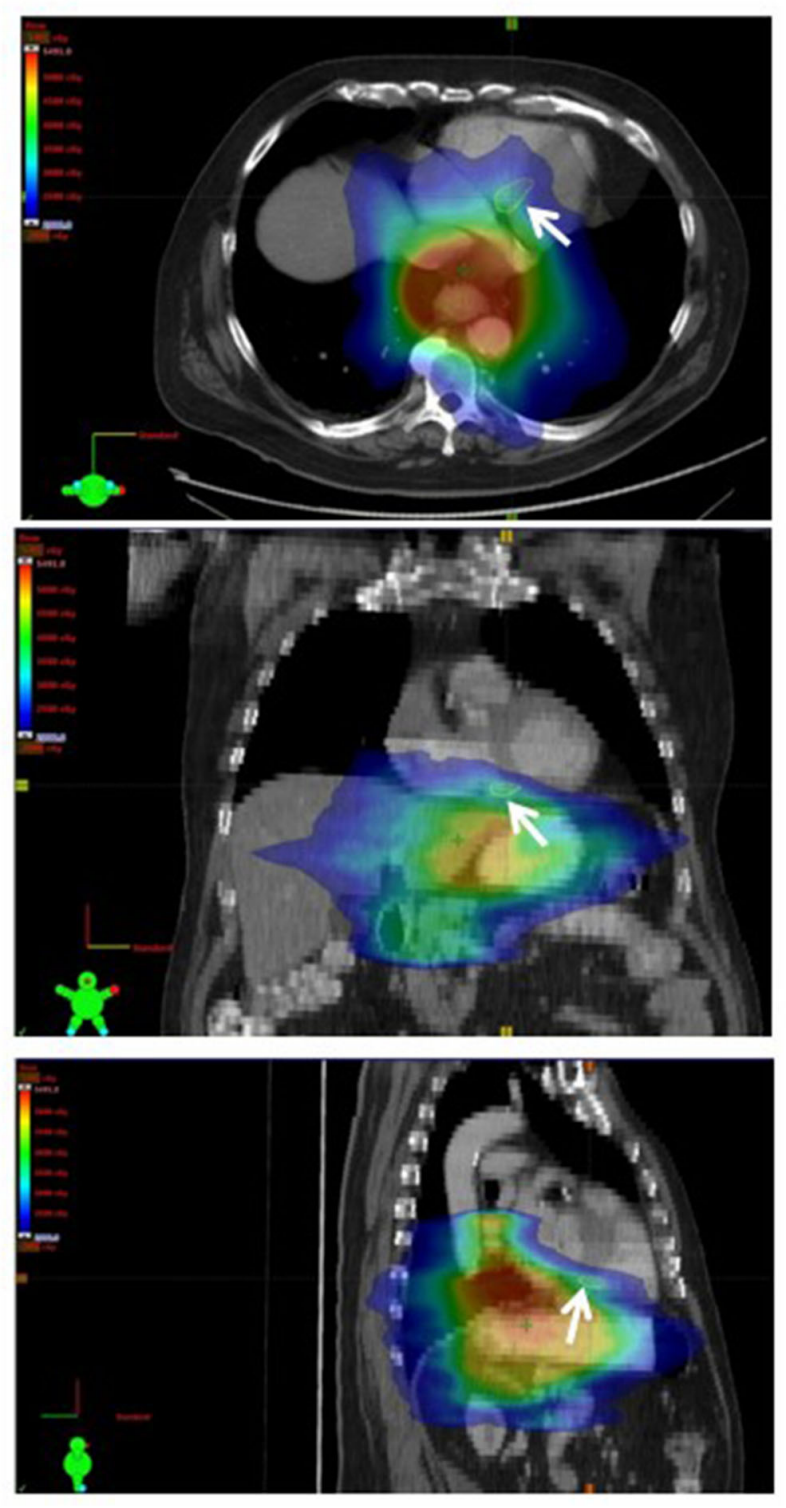
Representative treatment plan with axial (top), coronal (middle), and sagittal (bottom) CT images with color-washed dose distribution. The basal septal region is delineated (white arrow).

**Figure 2:**
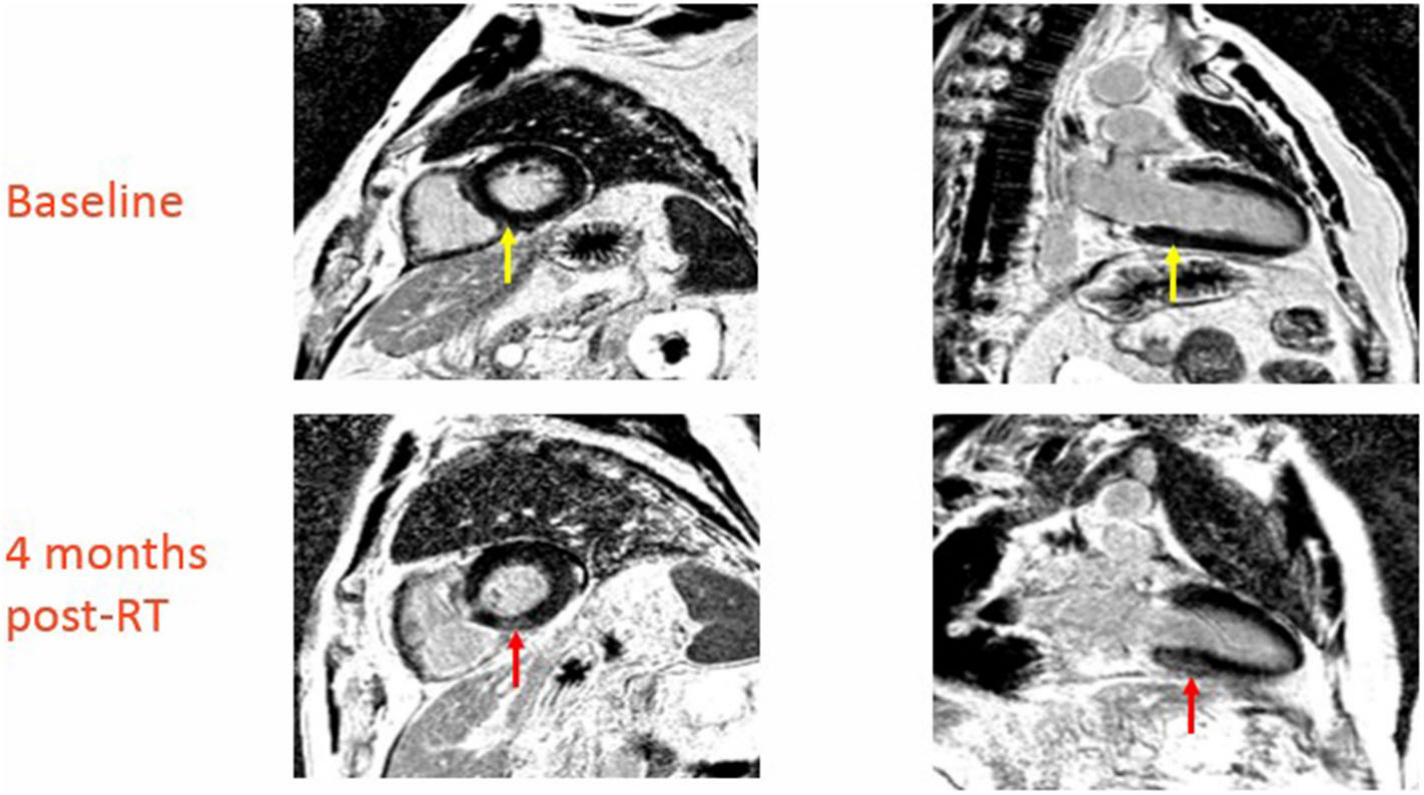
Baseline and 4-month post-RT CMR in patient with new CMR changes indicative of cardiac damage (red arrow in top images indicates area of late enhancement involving basal septum, yellow arrow in bottom images is the same region prior to radiation).

At baseline two out of 10 patients (20%) had pre-existing subepicardial fibrosis involving the basal or mid inferior/inferoseptal wall of the left ventricle, while the remaining patients (80%) did not have any evidence of pre-existing cardiac damage. Three out of 10 patients (30%) developed new structural findings of myocardial fibrosis and/or reversible ischemia involving the basal and mid inferior and inferoseptal walls. None of these three patients had prior evidence of cardiac damage. Figure 1 is a representative treatment plan with the corresponding involved myocardial segments contoured on axial, coronal, and sagittal CT images with overlying color-washed dose distribution. Representative CMR images demonstrating new areas of late gadolinium enhancement in the subepicardial and midwall portion of the myocardium consistent with fibrosis can be seen in Figure 2. In the three patients who showed signs of myocardial injury on MRI, the left ventricle end-systolic volume (LVESV) was significantly increased from baseline following radiation (*p* = 0.01). There was no significant difference in other quantitative end-points, including ejection fraction, in the three patients with myocardial damage or any quantitative end-points in the seven patients without myocardial damage. This data is shown in Table 2.

**Table 2:** Cardiac MRI pre and post-RT functional outcomes for patients with and without cardiac damage following chemoradiation.

	**With Structural Defects (*****n*** **= 3)**			**Without Structural Defects (*****n*** **= 7)**		
	**Pre-RT**	**Post-RT**	***p*-value**	**Pre-RT**	**Post-RT**	***p*-value**
LVEF (%)	68.7	67.0	0.5492	67.0	64.4	0.2456
LVEDV (mL)	144.0	161.3	0.1686	136.7	122.9	0.1694
LVESV (mL)	48.0	60.0	0.0091	46.1	46.0	0.9691
SV (mL)	96.3	101.3	0.5391	90.9	77.0	0.1165
CO (L/min)	7.1	7.0	0.8967	5.7	5.3	0.3628
Myocardial mass (g)	110.6	121.0	0.4842	117.4	109.8	0.1662

### Dosimetric Analysis

The basal or inferoseptal/inferior segments were contoured for each patient. Characteristics of radiation dose to the heart, left ventricle (LV) and involved segments for each patient are shown in Table 3. The average mean dose to the involved basal or inferoseptal/inferior segments was 29.5 Gy in patients with myocardial damage vs. 26.5 Gy in patients without damage (*p* = NS). In two out of three patients who had myocardial injury, the volume of involved basal or inferoseptal/inferior segments receiving 30 Gy was >60% of the total volume of those segments. Of note, the areas showing evidence of early radiation induced cardiac damage did not coincide with the segments of the heart receiving the highest dose of radiation.

**Table 3:** Dosimetric characteristics for patients with and without cardiac damage following chemoradiation.

**Patient ID**	**Septal wall** **mean dose (cGy)**	**Septal wall** **max dose (cGy)**	**Septal wall** **V30 (%)**	**Heart mean** **(cGy)**	**Heart V30** **(%)**	**LV mean** **(cGy)**	**LV max** **(cGy)**	**LV V30** **(%)**
1	33.2	44.4	71.7	12.6	10.0	15.1	50.2	13.0
2	30.4	48.8	51.4	20.6	16.3	18.0	52.6	7.6
3	24.9	49.9	21.0	20.3	15.1	18.3	54.4	7.5
Average (damaged)	*29.5*	*47.7*	*48.0*	*17.8*	*13.8*	*17.1*	*52.4*	*9.4*
4	30.2	45.4	46.8	26.7	30.2	25.3	52.8	28.2
5	37.4	53.9	67.3	20.6	25.5	31.8	54.3	47.7
6	20.4	39.5	4.7	22.2	20.0	20.6	50.0	11.9
7	20.2	50.9	8.1	18.8	14.3	17.4	54.4	9.0
8	2.9	37.0	0.7	6.1	10.0	3.7	52.7	5.0
9	31.1	51.6	45.3	23.2	23.6	26.5	54.1	27.1
10	30.6	56.3	38.3	27.1	28.2	25.1	55.1	25.7
11	39.2	52.6	86.9	14.9	17.3	22.3	52.8	30.2
Average (non-damaged)	*26.5*	*48.4*	*37.3*	*19.9*	*21.1*	*21.6*	*53.3*	*23.1*

### Serum Biomarker Analysis

**Figure 3:**
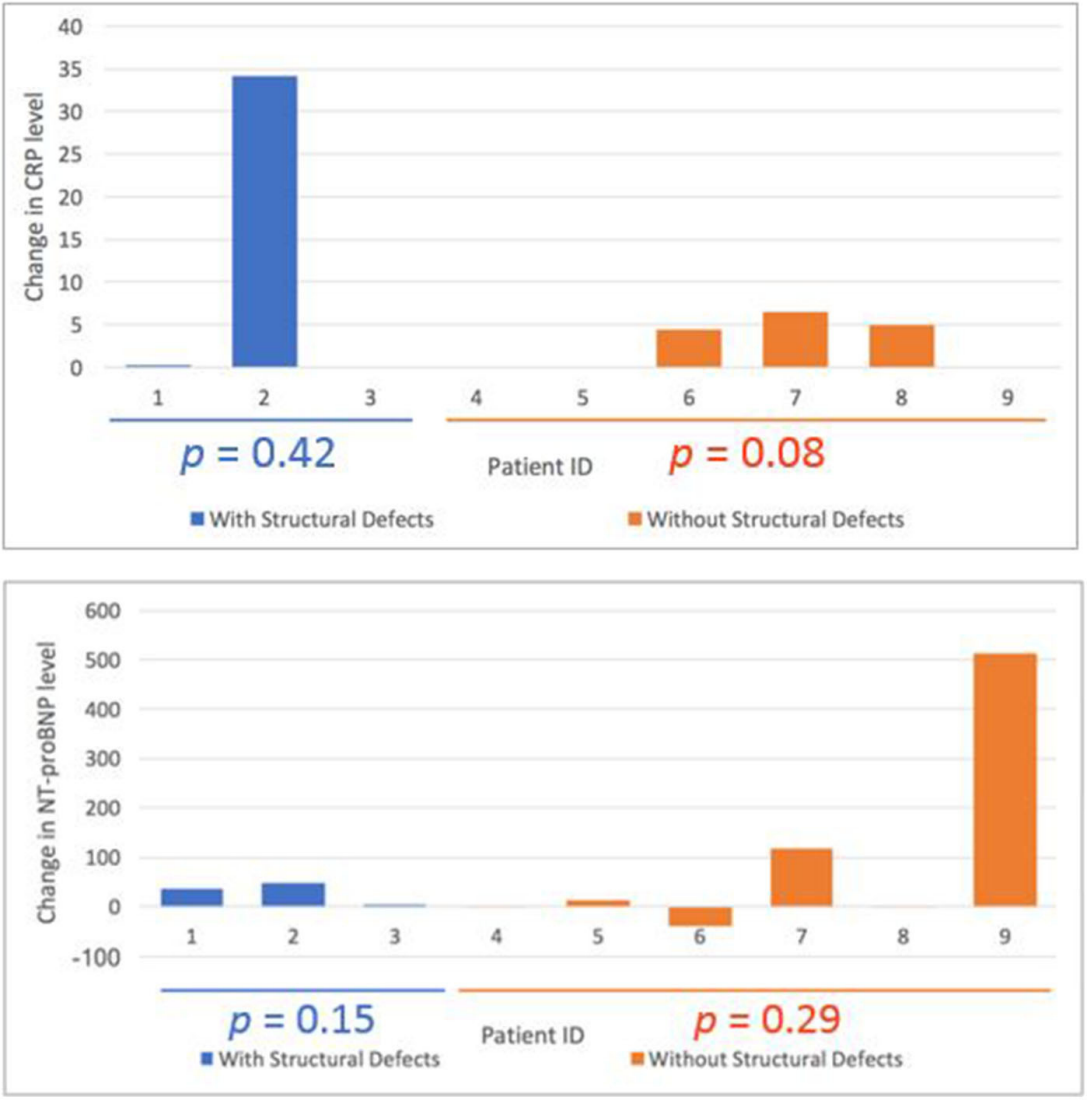
Change in pro-BNP and CRP in patients with structural damage (blue) vs. patients without structural damage (orange).

There were no significant changes in cardiac biomarkers when comparing pre-treatment and post treatment values amongst all patients. When stratified by those with MRI evidence of cardiac damage (*n* = 3) and those without (*n* = 7), there was still no significant difference in pre-post radiotherapy cardiac biomarkers (Figure 3).

## Discussion

Our study prospectively evaluated early cardiac toxicity following trimodality therapy for distal esophageal cancer using CMR to identify subclinical structural and functional heart damage. CMR allows for an unparalleled assessment of the myocardial and ventricular function. In order to eliminate the possibility of pre-existing cardiac damage, all patients were evaluated at baseline and 3–5 months following completion of chemoradiotherapy. Three of 10 patients developed new onset radiation-induced myocardial fibrosis or ischemia 4–5 months following therapy in the basal or mid inferior and inferoseptal segments. Of the patients who had CMR evidence of myocardial damage, the LVESV was significantly increased from baseline, though other markers of cardiac function including ejection fraction were not significantly altered following treatment. Given that this damage was seen in the same anatomic region of the heart in each patient, it is highly likely that it is associated with radiation therapy as opposed to systemic therapy or surgical resection. Our dosimetry analysis did not reveal a correlation between dose and heart damage, however, it is notable the involved areas of damage were not in the highest dose region of the heart and received a mean dose of 27.3 Gy across all patients. We did not find a correlation between serum biomarkers and cardiac toxicity.

There have been several analyses of the Surveillance, Epidemiology, and End Results (SEER) database that have correlated radiation for esophageal cancer with cardiac outcomes. Gharzai et al. analyzed 5,630 patients treated between 1973 and 2012 and found that patients treated with radiation had higher risk of cardiac death than those treated with surgery alone (12). Similarly, Frandsen et al. analyzed a larger cohort of 26,377 patients who received radiation as part of their therapy for esophageal cancer and noted an increased risk of heart disease specific survival when compared to those who did not receive radiation as part of their treatment for esophageal cancer. The absolute risk of heart disease related death in their analysis was 5.3% and 9.4% at 10 and 20-years, respectively. On multivariate analysis, RT remained predictive of heart disease related death with a hazard ratio of 1.46. Heart disease related death was noted to be detectable as early as 8 months from diagnosis (11). These studies both underscore that the cardiac effects of radiation have clinically significant outcomes that likely results in a survival detriment.

Umezawa et al. published one of the first reports using CMR to evaluate radiation induced myocardial damage in patients treated for esophageal cancer. They enrolled 24 patients who had maintained complete response to curative radiotherapy for esophageal cancer for more than 6 months with a median interval from completion of radiotherapy to CMR of 23.5 months (range 6–88 months). They noted late gadolinium enhancement corresponding to radiation fields in 15% of heart segments that received 40 Gy and 21% of heart segments that received 60 Gy (23). In contrast to our study in which CMR's were obtained both prospectively and 3–5 months post treatment, Umezawa et al. obtained post-treatment CMR over a wide range of time points at least 6 months after treatment. Additionally, they detected a correlation with higher dose and heart damage in segments throughout the heart.

Takagi et al. prospectively assessed changes in left ventricular function and tissue composition by comparing pre and post-treatment CMR in 24 patients with esophageal cancer treated with chemoradiation. They obtained CMR at baseline, 0.5 and 1.5 years and found that early imaging changes in myocardial tissue at 0.5 years preceded changes in LV stroke volume index that occurred at 1.5 years. The changes were most pronounced in the basal septum and occurred in areas that received higher radiation dose although the percent change from baseline did not correlate with radiation dose (24). Importantly, Takagi et al. and our study both demonstrate that the basal septum possibly has increased susceptibility to radiation induced damage. Notably we were able to detect this damage even earlier, as soon as 3 months following treatment with corresponding cardiac functional impairments. Other studies have demonstrated the impact of radiation dose on various segments of the heart including the left ventricle and the left mainstem coronary artery (25, 26). Although we were unable to detect a dose—volume relationship with the observed heart damage in the basal septum, we did find that in 2 of 3 patients with heart damage over 60% of the damaged segments received a dose of 30 Gy. Future research with additional patient numbers and longer follow up is required to determine whether the dose to individual or grouped segments should be considered in treatment planning.

In our patients who developed heart damage, we detected a significant increase in LVESV. LVESV has been shown to predict for the future development of heart failure in patients with ischemic heart disease. It is correlated with larger infarcts and is a major determinant of survival in patients after myocardial infarction (27). Future research with extended patient follow up will be necessary to fully characterize the impact of our finding of early increase in LVESV on the development of clinically apparent heart dysfunction.

We did not detect any significant changes in serum biomarkers when comparing pre and post-treatment levels nor when comparing those with cardiac damage to those without. While troponin I and CRP have been associated with changes in LVEF in patients undergoing chemotherapy for breast cancer, it is less well-reported with regards to early RT toxicity and there is similarly less consistent evidence to support BNP and NT-pro-BNP as an early biomarkers (18–20, 28–31). One consistent issue in reporting changes in biomarker values is the timing of collection and the thresholds for significant changes. Given the small sample size and lack of cardiac events in our group, it is not surprising that there was no detectable difference between groups.

Our study has several limitations. First, the relatively small sample size and currently short-term follow up. Longer follow-up will allow us to monitor for the natural history of the cardiac injury we identified and to assess for the development of clinically significant cardiac events and death. Second, while we hypothesize these findings are a result of radiation therapy given the similar anatomic location of the heart damage across affected patients, we cannot exclude the impact of other therapeutic modalities or patient related factors on our findings.

## Conclusion

Our study is the first to our knowledge to prospectively demonstrate radiation-associated structural and functional heart damage as early as 3 months following neoadjuvant chemoradiation for distal esophageal cancer. Given the early onset of this subclinical heart damage, strategies should be developed to identify patients at risk for future clinically significant heart toxicity. Although we did not find a correlative serum biomarker using those in routine clinical practice, novel biomarkers may be required to facilitate early detection of radiation associated heart damage. We identified that the basal or mid inferior and inferoseptal segments are potentially important cardiac substructures that demonstrate increased susceptibility to radiation damage; Further investigation to validate this finding and understand the dose—volume effects, as well as the role of advanced radiation therapy modalities in reducing cardiac dose, are needed.

## Data Availability Statement

The datasets generated for this study are available on request to the corresponding author.

## Ethics Statement

The studies involving human participants were reviewed and approved by Georgetown University IRB GU2015-1320. The patients/participants provided their written informed consent to participate in this study. Written informed consent was obtained from the individual(s) for the publication of any potentially identifiable images or data included in this article.

## Author Contributions

AMB: data analysis. CY: data collection. SK, PK, and MS: cardiac imaging. PB: cardiac imaging and contouring. AB: study enrollment and cardiac imaging. KU: content supervision and editing. All authors: contributed to the article and approved the submitted version.

## Conflict of Interest

The authors declare that the research was conducted in the absence of any commercial or financial relationships that could be construed as a potential conflict of interest.

## Abbreviations

CMR: cardiac magnetic resonance imaging
LGE: late gadolinium enhancement
LSESV: left ventricle end-systolic volume
LV: left ventricular
ASCO: American Society of Clinical Oncology
NCCN: National Comprehensive Cancer Network
ESMO: European Society for Medical Oncology
ASE: American Society of Echocardiography
EACVI: European Association of Cardiovascular Imaging
LVEF: left ventricular ejection fraction
BNP: B-type natriuretic peptides
NT-proBNP: N-terminal T-pro-BNP
CRP: C-reactive protein
GE: gastroesophogeal
ECOG: Eastern Cooperative Group
CHF: congestive heart failure
AV: advanced atrioventricular
IRB: Institutional review board
MR: magnetic resonance
AHA: American Heart Association
PBT: Proton Beam Therapy
IMRT: Intensity Modulated Radiation Therapy
CO: cardiac output
SV: systolic volume
SEER: Surveillance, Epidemiology, and End Results
IRB: Institutional review board.
